# Supplementary figures and images for: Comprehensive tissue-specific transcriptome profiling of pineapple (Ananas comosus) and building an eFP-browser for further study
Source: PeerJ. 2018 Dec 4;6:e6028. doi: 10.7717/peerj.6028 (PMC6284516; doi:10.7717/peerj.6028)

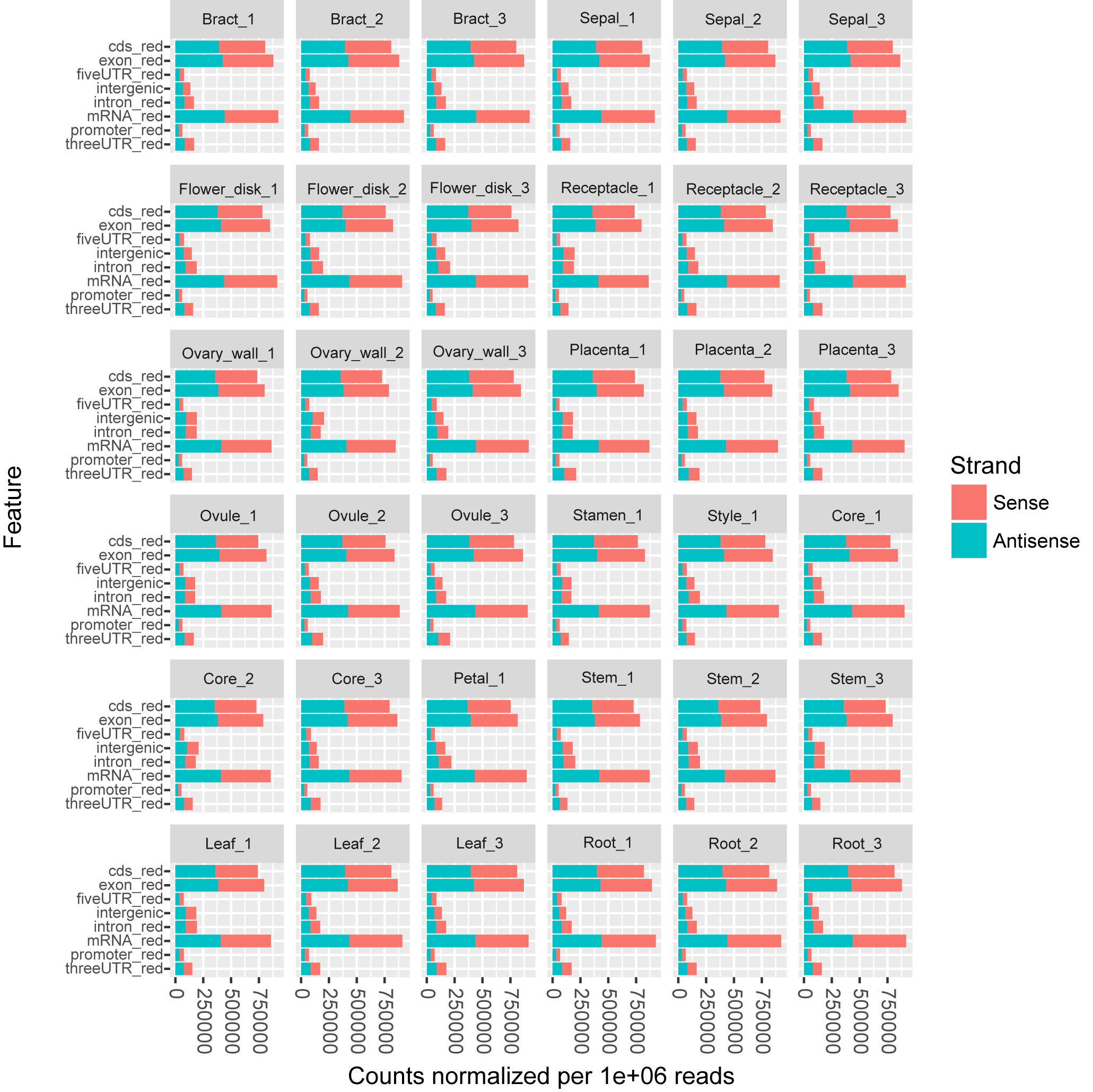

Supplement: Supplemental Information 1 [file peerj-06-6028-s001.jpg]

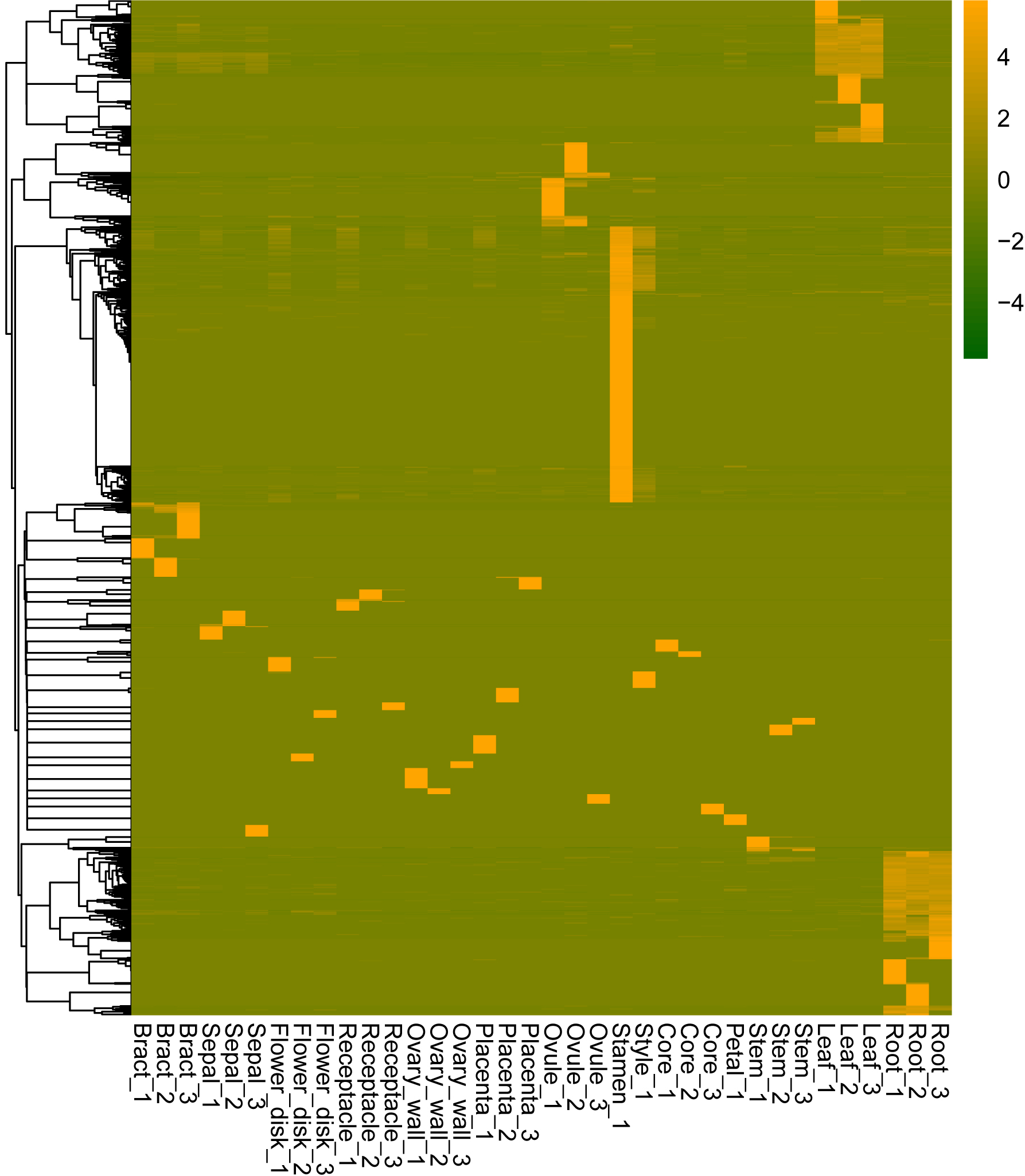

Supplement: Supplemental Information 2 [file peerj-06-6028-s002.jpg]

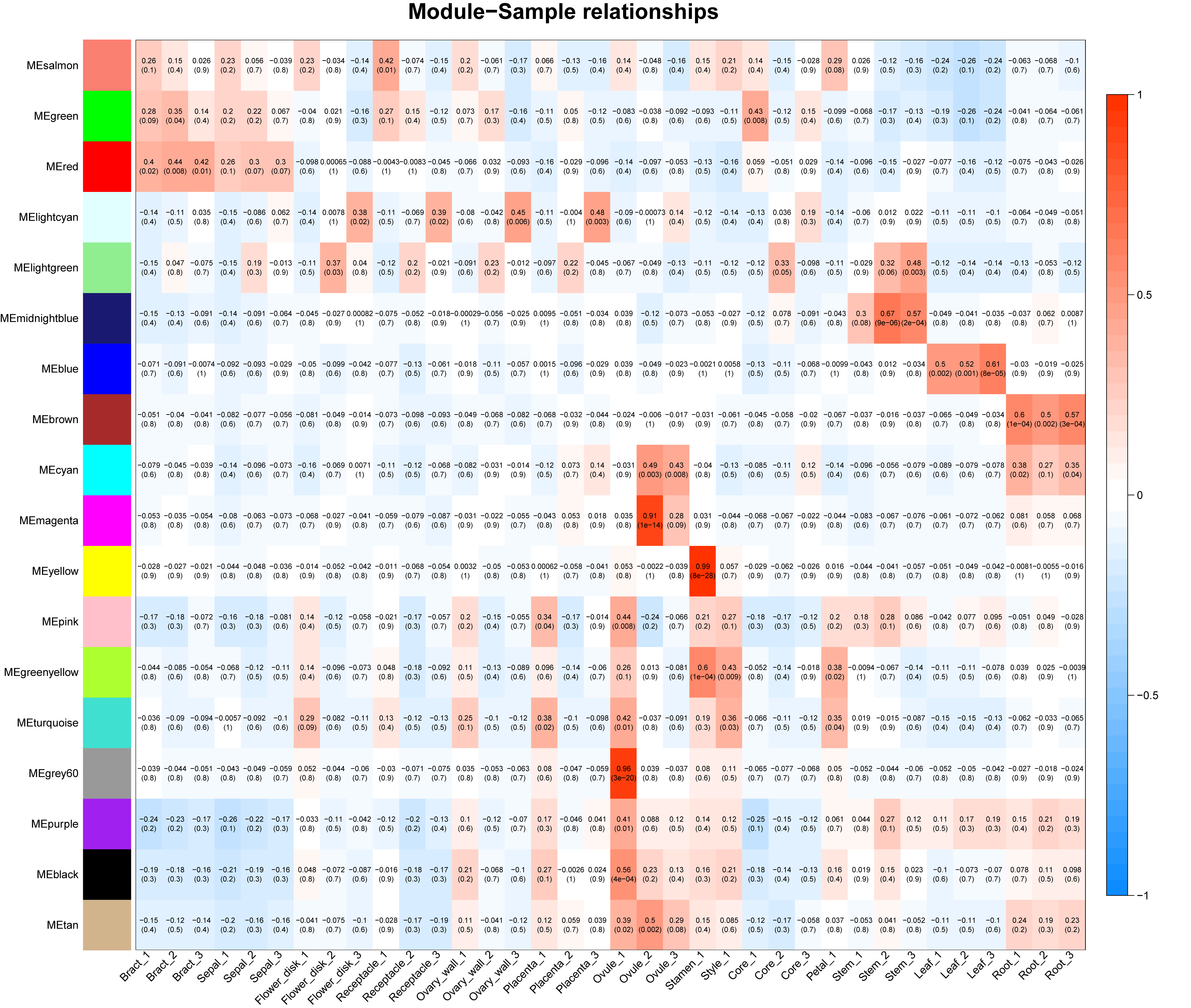

Supplement: Supplemental Information 3 [file peerj-06-6028-s003.jpg]

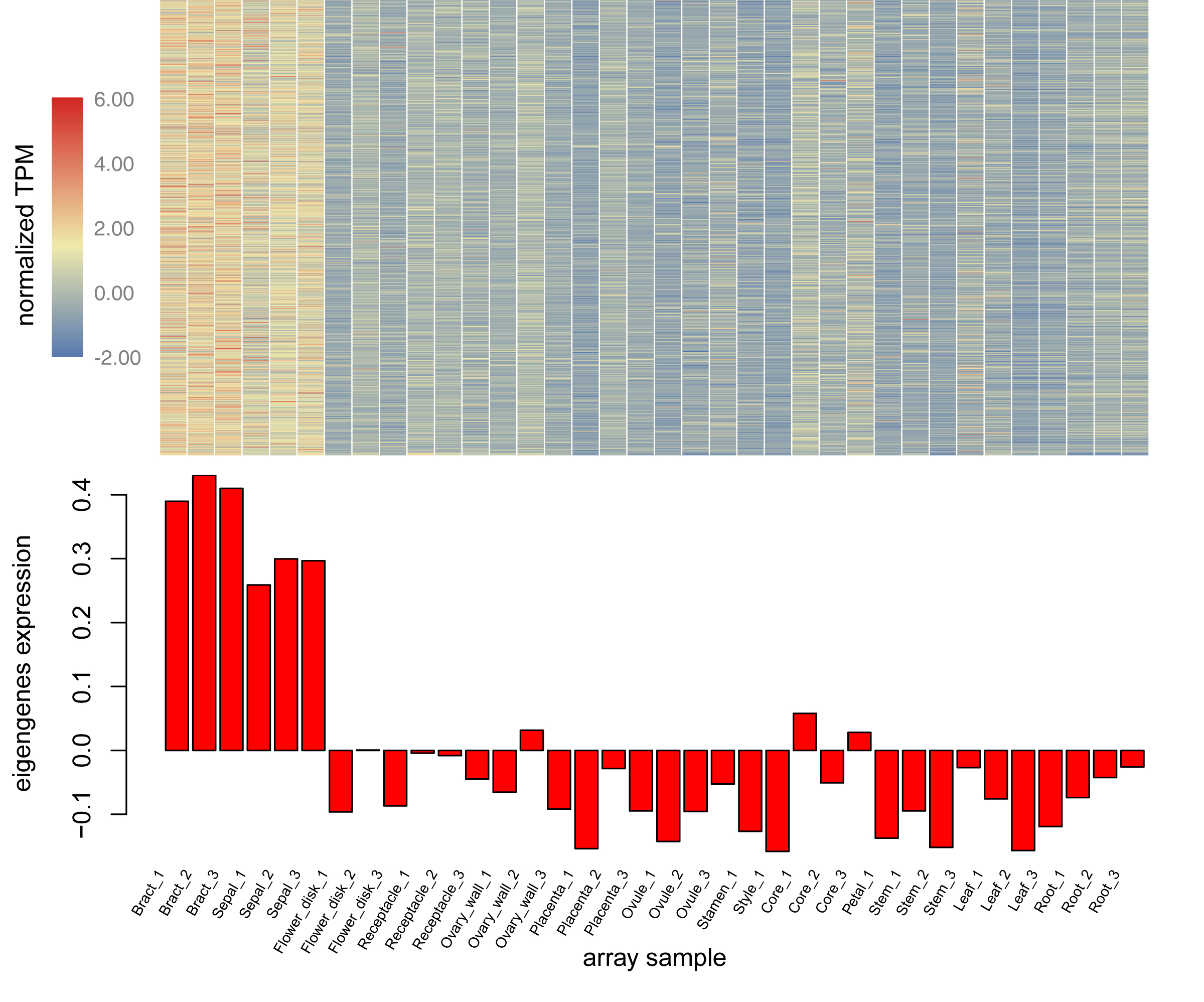

Supplement: Supplemental Information 4 [file peerj-06-6028-s004.jpg]

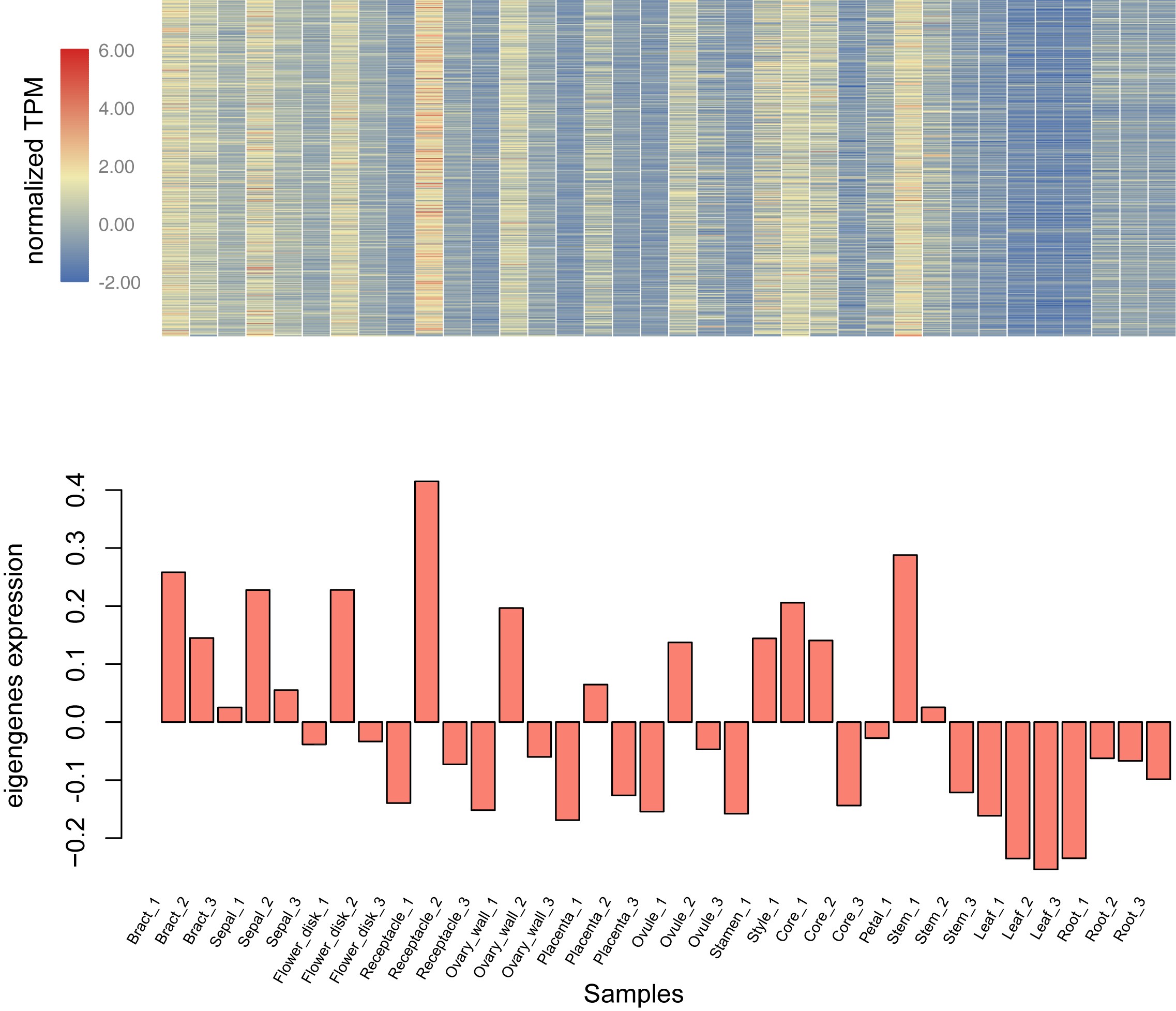

Supplement: Supplemental Information 5 [file peerj-06-6028-s005.jpg]

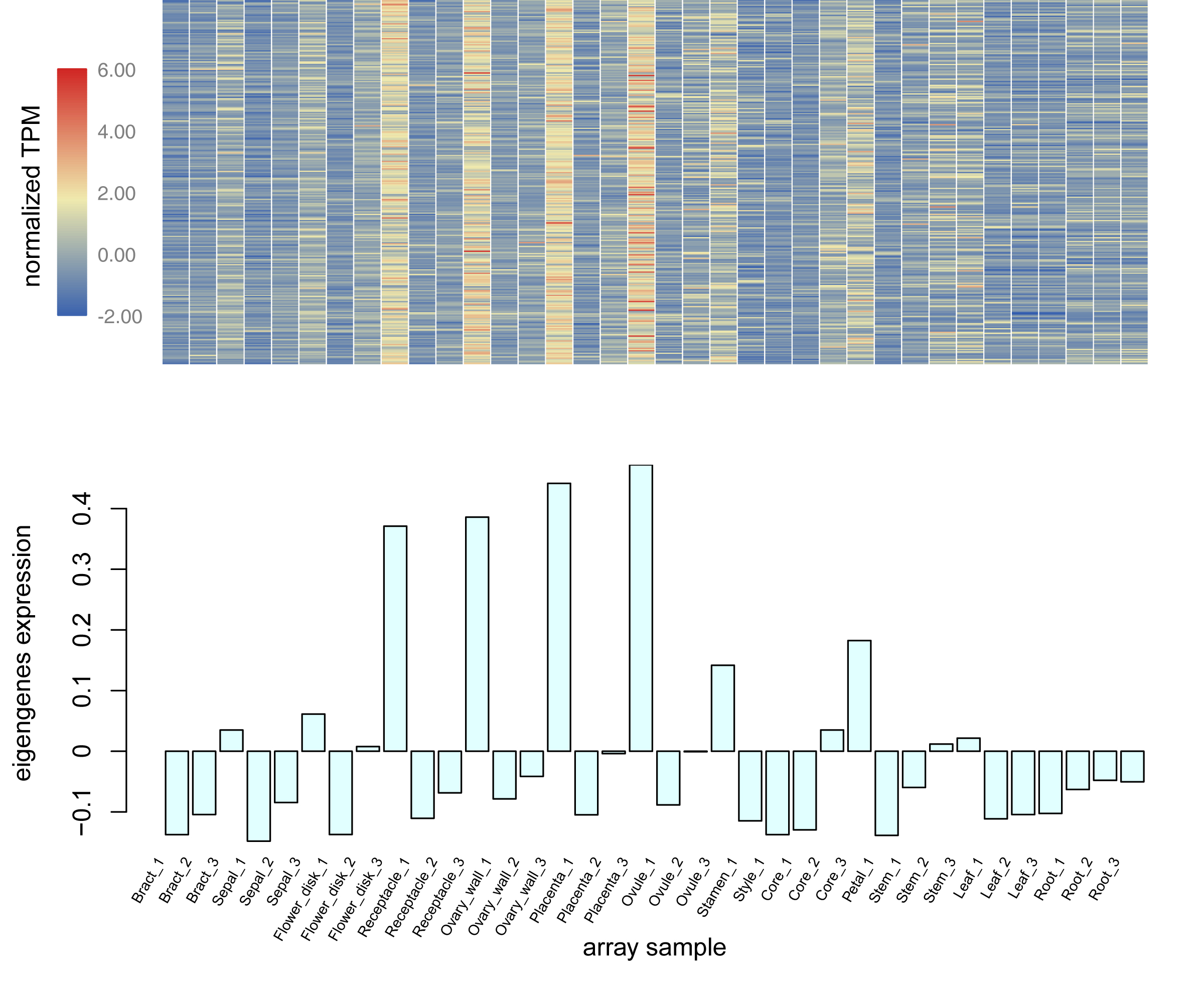

Supplement: Supplemental Information 6 [file peerj-06-6028-s006.jpg]
